# Urinary Metabolites of Volatile Organic Compounds in a Subset of East Palestine, Ohio Residents Five Months Following the Train Derailment Disaster

**DOI:** 10.1021/acs.est.6c01315

**Published:** 2026-09-04

**Authors:** Erin N. Haynes, Timothy J. Hilbert, Bela G. Nelson, Christine Kim, Misti Allison, Nicholas Peraino, Candace Brancato, Kristen McQuerry, Judy Westrick

**Affiliations:** † 4530 University of Kentucky, College of Public Health, Lexington, Kentucky 40536, United States; ‡ 2954 Wayne State University, Lumigen Instrument Center, Detroit, Michigan 48201, United States

**Keywords:** disaster response research, community-engaged research, report-back, metabolites, vinyl chloride, acrylate, HEMA

## Abstract

The February 2023 train derailment and subsequent vent and burn of 129,000 gallons of vinyl chloride in East Palestine, Ohio, raised a high level of concern about exposure to hazardous chemicals such as volatile organic compounds (VOCs) among residents. In this study, we measured urine metabolite levels of eight (8) VOCs collected five (5) months after the incident from 19 (19) nonsmoking East Palestine residents living within approximately one mile from the derailment site and compared them with levels from a comparison community and 2015–2016 National Health and Nutrition Examination Survey (NHANES) data. The samples were analyzed with a Thermo Altis LC–MS–MS system. Nearly 75% of East Palestine participants had detectable levels of a vinyl chloride metabolite, 2-hydroxyethylmercapturate (HEMA), whereas none of the comparison community participants had detectable levels and only 5% of NHANES participants had levels above the 3.0 ng/mL limit of detection (LOD). One East Palestine participant had a quantifiable level of the butyl acrylate metabolite, whereas none of the Marietta residents was above the LOD. The metabolite was not measured in the NHANES. The detection of metabolites of derailment-related chemicals supports the need for long-term follow-up of residents of this community and its surrounding area.

## Graphical abstract

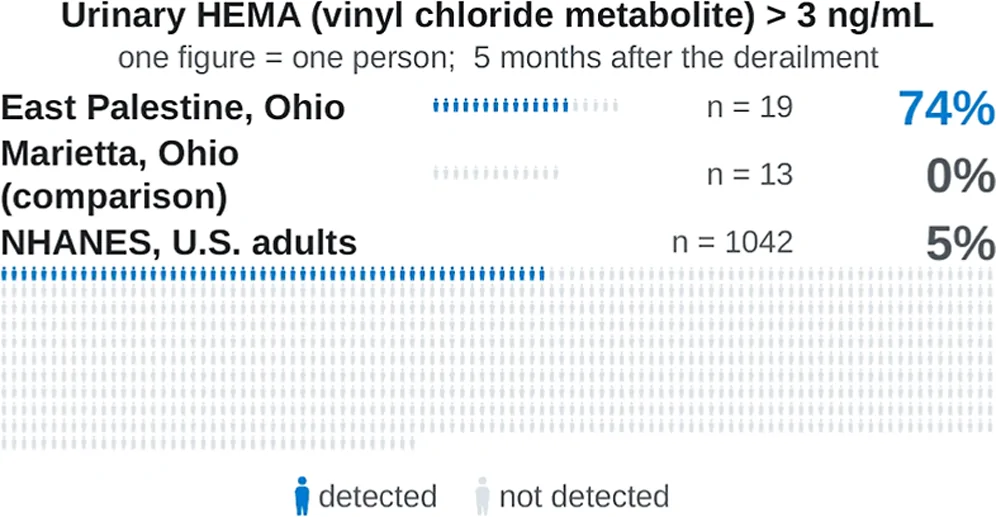

## Introduction

On the night of February 3, 2023, a Norfolk Southern train derailed in East Palestine, Ohio. This disaster resulted in a fire and subsequent vent and burn of five railway cars containing vinyl chloride, a volatile organic compound (VOC). According to the U.S. Environmental Protection Agency (EPA), 11 of the 52 derailed train cars were carrying hazardous materials, including vinyl chloride (5 cars containing 129,000 gallons (gal)), ethylene glycol monobutyl ether (EGBE, 1 car containing 30,110 gal), butyl acrylate (1 car containing 30,110 gal), ethylhexyl acrylate (1 car containing 25,800 gal.), isobutylene (1 car containing 30,110 gal), and two empty cars containing benzene residue.^1,2^ The cars containing butyl acrylate and EGBE were breached on impact and spilled their entire contents, while the car containing ethylhexyl acrylate partially spilled its contents.^3^ Exposure to VOCs such as these can result in eye, nose, and throat irritation, headaches, dizziness, nausea, and damage to the liver and kidneys.^4^ On the night of the train derailment, the village of East Palestine advised residents within a one-mile radius to evacuate, and a shelter-in-place order was issued for the remainder of the village of East Palestine.^5^ On February 4, 2023, release of “material” from pressure relief valves was reported for four of the five cars containing vinyl chloride,^3^ and on February 5, 2023, fearing an explosion and release of “deadly shrapnel”, the Ohio Emergency Management Agency (OEMA) issued an urgent evacuation notice urging residents living within one mile of the site to evacuate.^6^ On February 6, 2023, Ohio Governor DeWine and Pennsylvania Governor Josh Shapiro issued an expanded evacuation area to a one-mile by two-mile area,^7^ which was followed by the vent and burn of five (5) derailed cars containing vinyl chloride.^2^ This involved puncturing the railcars to release the vapor and liquid into a trench leading to a pit and then burning the spilled chemical.^3^ On 8^th^ February, 2 days following the “vent and burn” of 129,000 gallons of vinyl chloride, the OEMA announced that “evacuated residents in and around East Palestine can now safely return home”.^8^ Cleanup efforts began shortly after the derailment and continued through 2025.^9^ Cleanup activities included large-scale excavation of contaminated soil, removal and treatment of impacted water, and extensive sediment and surface water remediation in two impacted creeks, Sulphur Run and Leslie Run.^9^ Water sampling identified vinyl chloride in Sulphur Run and Leslie Run on February 8 and 10, 2023 and in a shallow monitoring well in July 2024.^10^ Vinyl chloride was identified at low levels in nearly 600 air samples in the months following the derailment.^11^ Soil sampling in April 2024 detected vinyl chloride at low levels in four samples.^12^ Based on the EPA Toxic Release Inventory (TRI), there are no other known nearby sources of vinyl chloride.^13^

In the months following the derailment, several East Palestine residents sought testing for vinyl chloride exposure.^14^ Urine samples were analyzed by a commercial laboratory for thiodiglycolic acid (TDGA), which is a metabolite of a broad range of physiological processes,^15^ one of which is the metabolism of vinyl chloride. TDGA has been used in epidemiologic research^16^ and is a reliable biomarker of vinyl chloride exposure when ambient levels are greater than 5 ppm;^17^ however, because it is typically found in urine of healthy persons and the potentially low levels of ambient vinyl chloride weeks after the derailment, it was not recommended for testing by the EPA^18^ and public health agencies.^19^ The lack of a suitable biomarker of exposure and the ability to provide biological samples for exposure measurements were frustrating for many community residents.

Thus, to address residents’ concerns regarding potentially lingering hazardous exposures related to the train derailment and vent and burn of vinyl chloride, a pilot study was conducted to measure urinary metabolite levels of targeted VOCs. The purpose of the pilot study was to determine if alternative vinyl chloride metabolites and other VOC metabolites related to the derailment could be measured in urine and, if so, how the levels compare to levels observed in another Appalachian Ohio community and a national comparison group. Pilot participants were invited through their participation in the University of Kentucky (UK) East Palestine Train Derailment Health Tracking Study,^20^ referred to as the Health Tracking Study, a community-engaged research study to assess and track residents’ health over time. Residents were eligible if they were 18 years of age or older and lived in Columbiana County or one of the five surrounding counties. A Community Advisory Board (CAB) was established^21^ to provide guidance and recommendations for the research and report-back of research results.

## Methods

Twenty (20) Health Tracking Study participants living within an approximately one-mile radius from the derailment site who were nonsmokers living in nonsmoking homes were invited to participate in the Biological Pilot Study that was conducted at the First United Presbyterian Church in East Palestine, Ohio. The pilot study was conducted on July 17–18, 2023. This study was approved by the UK Institutional Review Board (IRB no. 86158).

All participants provided informed consent. Blood was collected for quantification of dioxins and furans and immune function markers, and these methods and results are reported.^21,22^ Urine was collected into 100 mL sterile plastic cups via standard collection methods, taking care to avoid any contamination; 19 provided enough urine for measurement. The four (4) UK research staff provided urine samples upon arrival in East Palestine (July 16, 2023) and just prior to departure (July 18, 2023) in order to determine if there was a difference in urinary metabolites after being in the community for two (2) days. As lodging options are limited in East Palestine, each night the UK staff stayed about 15 miles from the study site. Urine samples were stored at 4 °C and shipped overnight to Wayne State University’s Lumigen Instrument Center.

In order to provide a comparison group, we enrolled participants from Marietta, Ohio, another Appalachian community in Ohio that is approximately 100 miles south of East Palestine. Marietta is of similar size as East Palestine and shares many socioeconomic characteristics (income, education, and race) as well as rural health risks (smoking rates, obesity, and healthcare access).^23^ Our team has longstanding community-engaged research partnerships in Marietta, which facilitated our recruitment of participants.^24^ The Marietta cohort urine samples were collected on November 27, 2023, from 20 participants using the same methodology as the East Palestine cohort. Thirteen (13) of the 20 were nonsmokers living in homes without other smokers; these 13 were used in our primary analyses.

All samples were analyzed by Wayne State University’s Lumigen Instrument Center for metabolites of VOCs including vinyl chloride, 2-hydroxyethylmercapturate (HEMA), acrolein, (3HPMA, 3-hydroxypropylmercapturate), CEMA­(*N*-acetyl-S-[2-carboxyethyl]-l-cysteine), butyl acrylate (butyl acrylate mercapturic acid), 2-ethylhexyl acrylate, (2-EHAMA, 2-ethylhexyl acrylate mercapturic acid), benzene, (PMA, phenylmercapturic acid)), toluene, (BMA (S-benzyl mercapturic acid)), ethylbenzene/styrene (phenyl gloxylic acid (PGA)), and xylene (methyl hippuric acid (MHA)). Vinyl chloride, butyl acrylate, and 2-ethylhexyl acrylate were of concern as they were released from the railcars that derailed, and acrolein was measured at elevated levels in the days following the derailment.^25^ Metabolites of BTEX (Benzene, Toluene, Ethylbenzene, Xylene) were analyzed to ensure proper laboratory procedures and for comparison with the other cohorts. The Lumigen Instrument Center developed novel specific measures for the acrylate metabolites.

## Metabolite Quantification

A 200 mM standard mixture was made by dilution of ethyl-acrylate (10.7 μL, 0.1 mmol), butyl-acrylate (12.2 μL, 0.1 mmol), 2-ethylhexyl-acrylate (20.8 μL, 0.1 mmol), and *N*-acetyl-cysteine (29.1 mg, 0.3 mmol) in 450 μL and left to sit at room temperature overnight. LC-HRMS–MS analysis shows the anticipated exact mass for [M-H]^−^ precursor mercapturic acid conjugates (ethyl: 262.0754, butyl: 290.1068, 2-ethylhexyl: 346.1693) with fragment ion 162.0234 (*N*-acetyl-cysteine).

An internal standard solution was prepared with BMA-d3, PGA-d5, MHA-d7, and PMA-d5 each at 100 ppb and used to standardize all samples, positive controls, and negative controls. A standard mixture at 1 ppm/10 μM was prepared for serial dilutions of 3-HPMA, HEMA, CEMA, PGA, MHA, PMA, BMA, BuAMA, and 2-EHAMA in methanol for spiking and calibrating. 100 μL of sample urine, control urine, or Sigmatrix urine was diluted with 100 μL of methanol and extracted with 25 μL of CHCl3. 100 μL of the aqueous top layer was diluted with 400 μL of LC–MS grade water for LC–MS–MS analysis. The 100 μL of methanol was in balance with the methanolic internal standard solution or spiking solution such that the total amount of methanol was 100 μL.

### Calibration and LOQ and Limit of Detection Determination

Calibrants were produced using Sigmatrix urine treated like samples at various spiking levels 1–500 ppb for commercial calibrants and 2.5–1700 ppb for the synthesized *N*-acetyl cysteine adducted acrylates. To account for matrix effects on analytes without an internal standard, the average recovery % versus the calibration was assessed and applied to the measurements. The limit of detection (LOD) and limit of quantification (LOQ) were determined by dividing the minimum LOD and LOQ in the Sigmatrix urine by this recovery.

Samples were analyzed with a Thermo Altis LC–MS–MS system eluting 0.1% formic acid in acetonitrile and water over a Waters BEH HSS-T3 100 mm × 2.1 mm × 1.8 μm column.

### Data Analysis

The National Health and Nutrition Examination Survey (NHANES) was used for a national comparison sample. NHANES is operated under the Center for Disease Control and Prevention’s (CDC) National Center for Health Statistics. The 2015–2016 data set provides national-level VOC metabolite data that can be used as a comparison for potentially exposed populations.^26^ We identified the metabolites of interest in NHANES 2015–2016 data and filtered to include only adult nonsmokers living in homes without other smokers. These NHANES data serve as U.S. background metabolite levels for nonsmokers. 2MHA and 34MHA, as well as 1DCV and 2DCV, were reported separately by NHANES and manually summed for our comparison. Metabolite levels from the Lumigen Instrument Center include values reported as < LOD and < Limit of Qualification (LOQ). Across all metabolites and cohorts, there were many < LOD results, but very few results reported as < LOQ. Values reported by the lab as < LOD or < LOQ were imputed as the LOD divided by the square root of 2 or LOQ divided by the square root of 2, respectively.^27,28^ Frequency of detection for metabolites includes results reported as < LOQ. The NHANES and the Lumigen Instrument Center each have different LOD for the metabolite analyses, so we include the LOD for comparison. We compared metabolite levels between our East Palestine participants (*n* = 19), our Marietta comparison group participants (*N* = 13), the UK staff (*n* = 4), and the NHANES 2015–2016 data set (*N* = 1042). Descriptive statistics, counts (*n*), percentages (%), and ranges were calculated for each metabolite. To illustrate differences in the metabolite levels among the groups, we generated boxplots for each metabolite.

### Report-Back of Data

Individual and group level data were prepared based on previous report-back strategies^21^ and shared with the study’s CAB for their input and feedback to ensure readability and comprehension of the report-back mechanism. The CAB also provided recommendations on sharing with the community-at-large.

## Results

The demographic characteristics of the East Palestine and Marietta, Ohio participants and NHANES 2015–2016 data are presented in Table 1. The mean age of our study participants at the time of enrollment was 55.4 years (standard deviation of 14.8 years), which was younger than the mean age of the Marietta participants (67.8 years). Similar to both the Marietta and NHANES comparison groups, sex was nearly evenly distributed between female (57.9%) and male (42.1%) participants. All East Palestine and Marietta participants were white, and the majority were non-Hispanic or Latino (94.7%). The NHANES cohort was more diverse in race. More East Palestine participants had earned a college degree (63.2%) than did the Marietta and NHANES cohorts. The majority (80%) of East Palestine and approximately 50% of participants from Marietta and NHANES had an annual household income of greater than $50,000. It should be noted that the NHANES income data were collected in 2015–2016; nearly 95% of East Palestine participants had a Body Mass Index (BMI) of 25 or higher, whereas Marietta and NHANES were 75% and 73%, respectively.

**Table 1:** Characteristics of East Palestine, Marietta, and NHANES 2015–2016 Participants

	East Palestine (*N* = 19)	Marietta (*N* = 13)	NHANES 2015–2016 (*N* = 1042)
**Sex, *n* (%)**			
male	8 (42.1)	6 (46.2)	477 (45.8)
female	11 (57.9)	7 (53.8)	565 (54.2)
**Age (years)**			
mean (SD)	55.4 (14.8)	67.8 (5.8)	50.4 (18.6)
Q1, median, Q3	44.5, 63, 64.5	64.5, 67, 69.5	35, 50, 65
minimum, maximum	31, 76	58, 78	18, 80
**Race, n (%)**			
white	19 (100)	13 (100)	698 (67.0)
**Ethnicity, n (%)**			
not hispanic or latino	18 (94.7)	12 (92.3)	679 (65.2)
**Income, n (%)**			
under $20,000	1 (6.7)	2 (16.7)	176 (18.4)
$20,000–$49,999	2 (13.3)	4 (33.3)	^a^319 (33.3)
$50,000–$74,999	3 (20)	0	^a^154 (16.1)
$75,000 and above	9 (60)	6 (50)	308 (32.2)
prefer not to answer	4	1	
**Education, *n* (%)**			
high school	4 (21)	3 (23)	429 (41.2)
some college	3 (15.8)	4 (30.8)	296 (28.4)
bachelor’s degree	8 (42.1)	4 (30.8)	^b^317 (30.4)
master’s degree	3 (15.8)	1 (7.7)	^b^
doctoral degree	1 (5.3)	1 (7.7)	^b^
**Body mass index (BMI), *n* (%)**			
<25 kg/m^2^	1 (5.3)	3 (25)	274 (26.5)
25–30 kg/m^2^	10 (52.6)	3 (25)	347 (33.6)
>30 kg/m^2^	8 (42.1)	6 (50)	412 (39.9)
**Smoking or vaping status, *n* (%)**			
never smoked or vaped	13 (68.4)	7 (53.8)	not available
previously smoked or vaped	6 (31.6)	6 (46.2)	not available
currently smoke or vape	0	0	0
**Anyone in household smoke or vape? *n* (%)**			
no	19 (100)	13 (100)	1042 (100)

a NHANES data grouped household income between $45 000 and $54 999. This group was split evenly into the $20 000 - $49 999 and $50 000 - $74 999 categories.

b Value includes all bachelor’s and graduate degrees.

Table 2 presents urinary metabolite levels across the three groups: East Palestine, Marietta, and NHANES. Nearly 75% of East Palestine participants had detectable levels of the vinyl chloride metabolite HEMA. In contrast, none of the comparison community participants had detectable levels of HEMA, and only 5% of NHANES participants had levels above an LOD of 3.0 ng/mL. One East Palestine participant had a measurable level of the butyl acrylate metabolite, whereas none of the Marietta residents had detectable levels. The NHANES did not measure this metabolite in the 2015–16 cohort.

**Table 2:** Number and Percent Detected and Range of Urine Metabolite Concentrations in Participants from East Palestine, Marietta, and the NHANES 2015–16 Cohorts

Parent chemical/metabolite, ng/mL	East Palestine (*N* = 19), n (%) detected, range	Marietta (*N* = 13), n (%) detected, range	NHANES 2015–16 (*N* = 1042), n (%) detected, range	East Palestine and Marietta LOD ng/mL	NHANES 2015–16 LOD ng/mL
vinyl chloride, ethylene oxide, and acrylonitrile/HEMA	14 (74), ND - 5.3	0 (0), ND	415 (40), ND - 12.8	3.0	0.79
			55 (5)[Table-fn t2fn2], ND - 12.8		3.0
acrolein/3HPMA	19 (100), 8.9–166.7	12 (92), ND - 95.8	966 (93), ND - 3510	6.0	13
acrolein/CEMA	19 (100), 10–204.8	12 (92), ND - 123.8	1037 (99.5), ND - 2610	5.0	6.96
benzene/PMA	1 (5), ND - 7.8	1 (8), ND - 10	466 (45), ND - 17.1	1.0	0.6
toluene/BMA	14 (74), ND - 7.6	13 (100), 1–68	1039 (99.7), ND - 1330	1.0	0.5
ethylbenzene and styrene/PGA	19 (100), >2–136.4	13 (100), 11.0–599.9	1031 (99), ND - 2400	2.0	12
xylene/MHA	19 (100), 3.3–131.6	13 (100), 5.2–160.6	1038 (99.6), ND - 16130	3.0	13
butyl acrylate/BuAMA	1 (5), ND - 11	0 (0), ND	NA	2.9	NA

LOD: limit of detection; ND: not detected; NA: not available.

a An LOD of 3 ng/mL would result in a reduction in the frequency of detection to 5%.

Four UK staff members also participated in the sampling process, and Table 3 presents the detected VOCs and their concentrations. Most metabolites showed a wider range of concentrations on day 2 compared to day 1. Of note, HEMA detections increased from two staff on day 1 to all four staff on day 2, with an increase in the upper range as well.

**Table 3:** Number, Percent Detected, and Range of Urine Metabolite Concentrations in UK Staff Members (*n* = 4) before and after Trip to East Palestine, Ohio

Parent chemical/metabolite, ng/mL	UK staff day 1 *n* (%) detected, range	UK staff day 2 *n* (%) detected, range
vinyl chloride, ethylene oxide, and acrylonitrile/HEMA	2 (50), ND - > 3	4 (100), >3–12
acrolein/3HPMA	4 (100), 9–72	4 (100), 20–100
acrolein/CEMA	4 (100), >5–45.7	4 (100), 13–67
benzene/PMA	0 (0), ND	0 (0), ND
toluene/BMA	2 (50), ND - 8.1	3 (75), ND - 7
ethylbenzene and styrene/PGA	4 (100), 12–64.5	4 (100), 14–200
xylene/MHA	4 (100), 13–56	4 (100), 3–95
butyl acrylate/BuAMA	0 (0), ND	0 (0), ND

ND: not detected; NA: not available.

Figure 1 presents the levels of analyzed VOCs across five groups: UK staff (day 1 and day 2), East Palestine and Marietta participants, and the NHANES 2015–2016 data.

**Figure 1:**
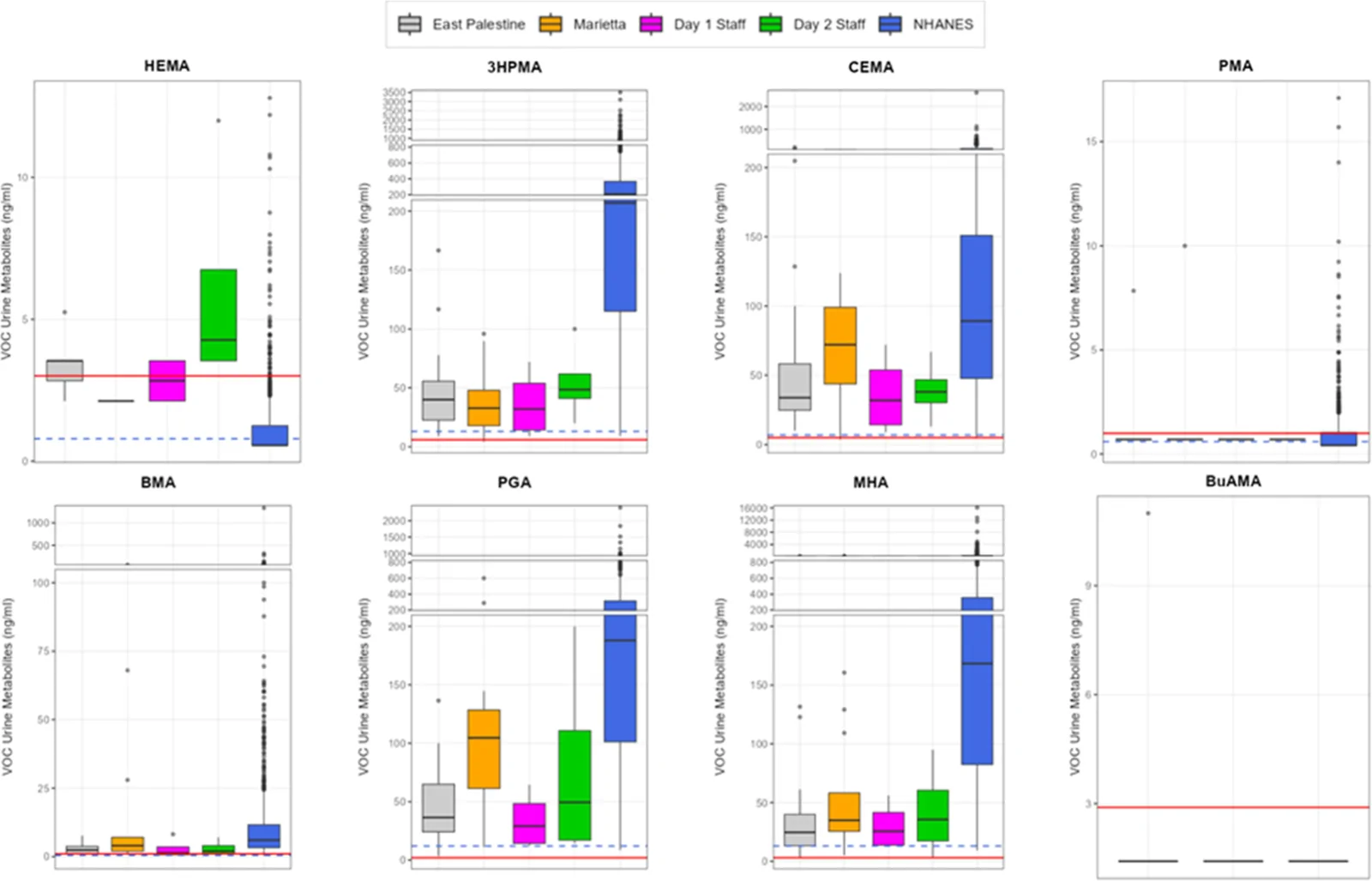
Concentrations of urine metabolites (ng/mL) in East Palestine, Marietta, UK Staff (day 1 and 2), and NHANES 2015–2016 data. Note: The solid red line represents the East Palestine, Marietta, and UK Staff LODs in ng/mL. The dotted blue line represents the NHANES 2015–16 LOD of ng/mL.

Table 4 presents a comparison of the metabolites between nonsmokers and the smoke-exposed (smokers or lives with a smoker) in the Marietta cohort. Most metabolites showed a narrower concentration range among nonsmokers compared to the smoke-exposed group, with the exception of BMA and PGA.

**Table 4:** Number, Percent Detected, and Range of Urine Metabolite Concentrations in Non-Smokers and Smoke-Exposed in the Marietta Cohort

Parent chemical/metabolite, ng/mL	Marietta nonsmokers (*n* = 13), *n* (%), range	Mmarietta smoke-exposed (*n* = 7), *n* (%), range
vinyl chloride, ethylene oxide, and acrylonitrile/HEMA	0 (0), ND	0 (0), ND
acrolein/3HPMA	12 (92), ND - 95.8	7 (100), 36.3–206.9
acrolein/CEMA	12 (92), ND - 123.8	7 (100), 18.5–191.0
benzene/PMA	1 (8), ND - 10	0 (0), ND
toluene/BMA	13 (100), 1–68	7 (100), 2–55
ethylbenzene and styrene/PGA	13 (100), 11.0–599.9	7 (100), 29.9–213.2
xylene/MHA	13 (100), 5.2–160.6	7 (100), 23.3–266.9

ND: not detected.

### Report-Back of Data

Individual level report-back included a brief description of the VOC and its representative urinary metabolite, along with the measurable level of the metabolite, if available. A comparison of how the participant’s individual values compared with the ranges found among other study participants and the NHANES study was also provided. If levels were detected, but not quantifiable (<LOQ), individuals were informed that the metabolite was detected in their sample, but at a level that was too low to be accurately measured. Participants were provided with group summary data for each metabolite. The CDC’s Agency for Toxic Substances and Disease Registry Toxicological Profiles^29^ were shared for additional information about each chemical. The report-back material drafts were shared with the CAB for feedback. Virtual meetings were held to allow for ease of feedback sharing.

## Discussion

Even months after the February 2023 train derailment in East Palestine, Ohio, residents had concerns about suspected lingering contamination in their communities. Our team worked with the community to determine whether levels of VOCs related to the derailment and vent and burn could be identified in residents living in close proximity to the derailment site. In this study, we compared urine VOC metabolite levels in East Palestine nonsmoking participants to those from a similar community in Marietta, Ohio, as well as data from the NHANES 2015–2016 data.

Nearly 75% of the East Palestine participants had a detectable level of the vinyl chloride metabolite HEMA, and one (1) of these had a quantifiable level. HEMA can also be formed from exposure to acrylonitrile or ethylene oxide; however, based on the EPA TRI database, there are no known sources of these chemicals near East Palestine. In addition, 12 of our 19 participants provided current occupational information, and no industrial exposures were reported. Approximately 40% of the NHANES 2015–2016 cohort had detectable levels of HEMA. The NHANES LOD for HEMA (0.79 ng/mL) is substantially lower than the LOD used by the Lumigen Instrument Center (3 ng/mL). When normalizing the LOD for both cohorts to 3 ng/mL, only 5% of NHANES participants had an HEMA level above 3 ng/mL, compared to 74% of our pilot study participants. In contrast, none of the Marietta participants had detectable HEMA levels. In the first sample, two of the staff (50%) had detectable levels of HEMA, while in the second sample all four staff (100%) had detectable levels of HEMA. Every UK staff member had an increase in the HEMA level between the first and second measurements.

Vinyl chloride was detected in only four East Palestine EPA soil samples. These samples were collected near the derailment site in April 2024 and, although they were low levels, the levels exceeded the incident-specific levels, and the soil was removed. The EPA stated that there was no risk to the community from these detections and targeted cleanups.^12^ However, this demonstrates the presence of vinyl chloride in the community over a year after the derailment. A potential mechanism for the presence of vinyl chloride in air, water, and soil samples months following the derailment may be that some of the vinyl chloride released during the derailment from the pressure relief valves or during the “vent and burn” resided in the creek beds until being redistributed by cleanup efforts. Vinyl chloride can persist in wet, anaerobic sediments for much longer than in air, surface water, or soil.^30^ In addition, VOCs can also be harbored in fabrics such as clothing, carpeting, and curtains.^31^

Butyl acrylate exposure is not common. The East Palestine participant had a quantifiable level of the butyl acrylate metabolite. While NHANES did not measure this metabolite, none of the Marietta residents or the UK staff had a detectable level of this metabolite. The railroad car containing butyl acrylate spilled its entire contents in East Palestine, Ohio.

UK staff also saw concentration increases in 3HPMA, PGA, and MHA from day 1 to day 2. For the VOC metabolites other than HEMA, UK staff concentrations were similar to those among the East Palestine participants.

Most of the metabolites measured in UK staff and East Palestine participants had lower median concentrations compared to both Marietta participants and the NHANES 2015–2016 data, including BTEX. For example, the median for the benzene metabolite BMA in the East Palestine participants was 2.9 ng/mL, compared to 4 ng/mL in the Marietta group and 6.1 ng/mL in the NHANES group (Table S1). There is also the possibility that VOCs outside the scope of this study are still present in the community.^25,32^

VOCs with high lipid solubility, such as benzene and toluene, can persist longer in the body due to accumulation in fatty tissues.^33^ To explore this, we assessed the relationship between BMI and measured urine metabolites and found that CEMA was significantly correlated with BMI in both the NHANES and East Palestine participants (data not shown). This association was not observed in the Marietta comparison groups. This finding also aligns with a previous study that reported a positive association between CEMA and obesity, abdominal obesity, as well as waist circumference.^34^

EPA air sampling data^11^ from July 15–18, 2023, our study time frame, did not identify any vinyl chloride, butyl acrylate, ethylhexyl acrylate, or styrene above the EPA reporting level. All BTEX chemicals were identified in EPA air sampling over those days but at levels well below the EPA reference concentration. The EPA did not test for acrolein or ethyl acrylate in July 2023.

Following the derailment, acrolein was identified in environmental sampling, but our study did not detect elevated levels of its metabolites; levels were similar to the comparison community and lower than NHANES. The railcar containing ethylhexyl acrylate partially released its contents during the derailment, and the metabolite of ethylhexyl acrylate was not detected in any of our cohorts’ urine samples. This could be related to the LOD or the amount released. NHANES did not test for ethylhexyl acrylate.

There is limited literature regarding metabolites of our chemicals of primary interest—vinyl chloride, acrolein, and acrylates—and even less without a creatinine adjustment. However, reference ranges for nonsmoking populations are available. In nonsmokers, Ding et al. found CEMA, 3HPMA, HEMA, and PMA ranges of ND – 94 ng/mL, ND-123 μg/L, ND-1.44 ng/mL, and ND-0.26 ng/mL, respectively.^35^ East Palestine residents had higher levels for each of these metabolites: 10–204 ng/mL, 8.9–166.7 ng/mL, ND-5.3 ng/mL, and ND-7.8 ng/mL, respectively. While there are medical guidelines for some VOC exposures,^36,37^ the corresponding threshold values of urinary metabolites have not been established. Further research could investigate health thresholds for VOC urine metabolites.

This research also involved the development of a methodology for testing acrylate metabolites by Wayne State University. To our knowledge, other research on levels of these metabolites has not been published, and these metabolites were not included in the NHANES study. This work can provide reference values for acrylate metabolites for future research.

The sizes of the East Palestine and Marietta groups are small; however, they served the purpose of pilot exploration of urinary VOC metabolite levels after the disaster in response to community concerns about suspected lingering exposures. Although a small sample, all participants were nonsmokers who lived in nonsmoking homes, which minimizes any potential confounding effects of smoking. In addition, our participants resided approximately one mile or less from the derailment site, and all but two had been living in their home after the evacuation order was lifted. To our knowledge, this was the earliest and possibly the only measure of VOC urinary metabolites following the disaster. This was feasible through strong community engagement throughout the conduct of the research. The detection of metabolites of derailment-related chemicals supports the need for long-term health follow-up of residents of this community and its surrounding area.

## Supplementary Material



## Notes

Table S1: Medians for the analyzed VOC urine metabolites in East Palestine, Marietta, UK staff (Day 1 and 2), and NHANES 2015-2016 data (DOCX)

## References

[1] Environmental Protection Agency (EPA) . East Palestine Train Derailment Site East Palestine, Columbiana County, Ohio. In Unilateral Administrative Order for Removal Actions; United States Environmental Protection Agency, 2023; Vol. 2023.

[2] Environmental Protection Agency (EPA) . TRAIN 32N- EAST PALESTINE. 2023. https://www.epa.gov/system/files/documents/2023-02/TRAIN%2032N%20-%20EAST%20PALESTINE%20-%20derail%20list%20Norfolk%20Southern%20document.pdf(accessed Nov 11, 2025).

[3] National Transportation Safety Board (NTSB) Railroad Investigation Report RIR-24–05 , Norfolk Southern Railway Derailment Hazardous Materials Release – East Palestine, Ohio February 3, 2023. https://www.ntsb.gov/investigations/AccidentReports/Reports/RIR2405%20CORRECTED.pdf (accessed May 28, 2026).

[4] Environmental Protection Agency (EPA) . Volatile Organic Compounds. Impact on Indoor Air Quality. 2025. https://www.epa.gov/indoor-air-quality-iaq/volatile-organic-compounds-impact-indoor-air-quality (accessed Nov 11, 2025).

[5] CBS News . 50-car train derailment in Ohio causes massive fire, evacuations. 2023. https://www.cbsnews.com/news/ohio-train-derailment-causes-big-fire-evacuations/(accessed May 28, 2026).

[6] Linder, L. , Guay, J. , Smith, B. , DeRose, C. East Palestine Train Derailment: Evacuations ordered with rail car at risk for explosion following massive fire. 2023. https://www.cbsnews.com/pittsburgh/news/train-derailment-massive-fire-east-palestine-ohio-ntsb-investigation-explosion-evacuations/ (accessed Jan 22, 2026).

[7] Ohio Emergency Management Agency . East Palestine Update: Evacuation Area Extended, Controlled Release of Rail Car Contents Planned for 3:30 P.M. 2023. https://ema.ohio.gov/media-publications/news/dewine-020623 (accessed Nov 11, 2025).

[8] Office of the Governor of Ohio . East Palestine Update: Residents Can Safely Return Home. 2023. https://governor.ohio.gov/media/news-and-media/east-palestine-update-residents-can-safely-return-home-02082023 (accessed Nov 11, 2025).

[9] Environmental Protection Agency (EPA) . East Palestine, Ohio Train Derailment. 2026.https://www.epa.gov/east-palestine-oh-train-derailment (accessed Nov 11, 2025).

[10] Environmental Protection Agency (EPA) . East Palestine, Ohio Train Derailment Water Sampling Data. 2026. https://www.epa.gov/east-palestine-oh-train-derailment/water-sampling-data (accessed May 28, 2026).

[11] Environmental Protection Agency (EPA) . Air Sampling Data. 2025. https://www.epa.gov/east-palestine-oh-train-derailment/air-sampling-data (accessed Nov 11, 2025).

[12] Environmental Protection Agency (EPA) . Newsletter: East Palestine Train Derailment Response, 7–8-2024. 2025. https://www.epa.gov/east-palestine-oh-train-derailment/newsletter-east-palestine-train-derailment-response-7-8-2024 (accessed Nov 11, 2025).

[13] Environmental Protection Agency (EPA) . Toxic Release Inventory (TRI) Program. https://www.epa.gov/toxics-release-inventory-tri-program (accessed May 28, 2026).

[14] S., Elverd The Review. VC metabolite prevalent in East Palestine urine samples. 2025. https://www.reviewonline.com/news/local-news/2025/11/vc-metabolite-prevalent-in-east-palestine-urine-samples/(accessed May 28, 2026).

[15] T., Navrátil , Petr, M. ; Z., Šenholdová , K., Přistoupilová , T. I., Přistoupil , M., Heyrovský , D., Pelclová , E., Kohlíková . Diagnostic Significance of Urinary Thiodiglycolic Acid as a Possible Tool for Studying the Role of Vitamins B12 and Folates in the Metabolism of Thiolic Substances. Physiol. Res. 56: 113–122, 2007. https://www.biomed.cas.cz/physiolres/pdf/56/56_113.pdf (accessed May 28, 2026). 10.33549/physiolres.930902 16497089

[16] C.-W., Wang , H.-Yi., Chuang , K.-W., Liao , M.-L., Yu , C.-Y., Dai , W.-T., Chang , C.-H., Tsai , H.-C., Chiang , Po-C., Huang Urinary thiodiglycolic acid is associated with increased risk of non-alcoholic fatty liver disease in children living near a petrochemical complex. Environ. Int., 131, 2019, 104978, ISSN 0160–4120, DOI: 10.1016/j.envint.2019.104978. https://www.sciencedirect.com/science/article/pii/S0160412019308979(accessed May 28, 2026). 31325714

[17] Cheng, T. J. , Huang, Y. F. , Ma, Y. C. Urinary thiodiglycolic acid levels for vinyl chloride monomer-exposed polyvinyl chloride workers. J. Occup Environ. Med. 2001;43(11):934–938. doi: 10.1097/00043764-200111000-00002. PMID: 11725332. https://pubmed.ncbi.nlm.nih.gov/11725332/(accessed May 28, 2026). 11725332

[18] Environmental Protection Agency (EPA) . Vinyl Chloride and Health FAQ Document. May 31, 2023. https://www.epa.gov/system/files/documents/2023-06/Vinyl-Chloride-and-Health-FAQ-Document-5-31-508.pdf (accessed May 28, 2026).

[19] T., Morgan ABC NEWS 5 Cleveland. Urine tests from East Palestine residents unreliable for vinyl chloride exposure, ODH Director Says. 2023. https://www.news5cleveland.com/news/local-news/we-follow-through/urine-tests-from-east-palestine-residents-unreliable-for-vinyl-chloride-exposure-odh-director-says (accessed May 28, 2026).

[20] Haynes, E. East Palestine Train Derailment Health Tracking Study. 2023. https://cph.uky.edu/research/projects/east-palestine-train-derailment-health-tracking-study (accessed Nov 25, 2025).

[21] Haynes E.
N., Eskenazi B., Hilbert T. J., Brancato C., Holland N., Kim C., Calafat A. M., Jones R., Davis M., Birnbaum L. S. (2024). Serum Dioxin Levels in a Subset of Participants of the East Palestine,
Ohio Train Derailment Health Tracking Study. Environ. Sci. Technol. Lett..

[22] Wagner S. B., Hemati H., Gandhapudi S., Hilbert T. J., Brancato C., Haynes E. N., Messaoudi I. (2026). Characterizing
immune perturbations
in peripheral blood following the East Palestine, Ohio train derailment. J. Exposure Sci. Environ. Epidemiol..

[23] United States Census Bureau . Explore Census Data. https://data.census.gov/(accessed May 28, 2026).

[24] Haynes, E. N. , Beidler, C. , Wittberg, R. , Meloncon, L. , Parin, M. , Kopras, E. J. , Succop, P. , Dietrich, K. N. Developing a bidirectional academic-community partnership with an Appalachian-American community for environmental health research and risk communication. Environ. Health Perspect. 2011 Oct;119(10):1364–1372. doi: 10.1289/ehp.1003164. Epub 2011 Jun 16. PMID: 21680278; PMCID: PMC3230433. https://pmc.ncbi.nlm.nih.gov/articles/PMC3230433/ (accessed May 28, 2026). PMC3230433 21680278

[25] Oladeji O., Saitas M., Mustapha T., Johnson N. M., Chiu W. A., Rusyn I., Robinson A. L., Presto A. A. (2023). Air Pollutant Patterns
and Human Health Risk following the East Palestine, Ohio, Train Derailment. Environ. Sci. Technol. Lett..

[26] Centers for Disease Control and Prevention (CDC) . About NHANES. 2024. https://www.cdc.gov/nchs/nhanes/about/index.html (accessed Nov 25, 2025).

[27] Croghan, C. , Egeghy, P. P. 2003. Methods of Dealing with Values Below the Limit of Detection Using SAS. https://cfpub.epa.gov/si/si_public_record_report.cfm?Lab=NERL&dirEntryId=64046 (accessed May 28, 2026).

[28] Lubin J. H., Colt J. S., Camann D., Davis S., Cerhan J. R., Severson R. K. (2004). Epidemiologic
evaluation of measurement data
in the presence of detection limits. Environ.
Health Perspect..

[29] Agency for Toxic Substances and Disease Registry (ATSDR) . Toxicological Profiles. 2025. https://www.atsdr.cdc.gov/toxicological-profiles/about/index.html (accessed Nov 25, 2025).

[30] Agency for Toxic Substances and Disease Registry (ATSDR) . 2026. Toxicological Profile for Vinyl Chloride https://www.ncbi.nlm.nih.gov/books/NBK601940/ (accessed May 28, 2026). 38498642

[31] Huynh H. N., Ditto J. C., Yu J., Link M. F., Poppendieck D., Farmer D. K., Vance M. E., Abbatt J. P. D. (2024). VOC emission
rates from an indoor surface using a flux chamber and PTR-MS. Atmos. Environ..

[32] Saitas M., Mustapha T., Vitucci E., Oladeji O., Tsai H. D., Cannon C., Rusyn I., Presto A. A., Chiu W. A., Johnson N. M. (2025). Mobile air monitoring
to identify volatile organic
compound distributions and potential hazard during the remediation
of the East Palestine, Ohio train derailment. Environ. Monit. Assess..

[33] a National Research Council . Systemic Exposures to Volatile Organic Compounds and Factors Influencing Susceptibility to Their Effects. National Academies Press (US), 2009. https://www.ncbi.nlm.nih.gov/books/NBK215288/ (accessed Nov 25, 2025)From NLM Medline

[34] Lei T., Qian H., Yang J., Hu Y. (2023). The association analysis
between exposure to volatile organic chemicals and obesity in the
general USA population: A cross-sectional study from NHANES program. Chemosphere.

[35] Ding Y. S., Blount B. C., Valentin-Blasini L., Applewhite H. S., Xia Y., Watson C. H., Ashley D. L. (2009). Simultaneous
determination of six
mercapturic acid metabolites of volatile organic compounds in human
urine. Chem. Res. Toxicol..

[36] Agency for Toxic Substances and Disease Registry (ATSDR) . Medical Management Guidelines for Vinyl Chloride. 2014. https://wwwn.cdc.gov/TSP/MMG/MMGDetails.aspx?mmgid=278&toxid=51 (accessed Nov 25, 2025).

[37] Centers for Disease Control and Prevention (CDC) . Benzene: Systemic Agent. 2011. https://www.cdc.gov/niosh/ershdb/emergencyresponsecard_29750032.html (Agency for Toxic Substances and Disease Registry (ATSDR). Medical Management Guidelines for Acrolein. 2014. http://medbox.iiab.me/modules/en-cdc/www.atsdr.cdc.gov/MMG/MMG.asp_id=552&tid=102 (accessed Nov 25, 2025).

