# Supplementary material for: Urinary Metabolites of Volatile Organic Compounds in a Subset of East Palestine, Ohio Residents Five Months Following the Train Derailment Disaster
Source: Environ Sci Technol. 2026 Sep 4;60(38):26953–60. doi: 10.1021/acs.est.6c01315 (PMC13629621; doi:10.1021/acs.est.6c01315)
Supplement: Supplementary file 1 [file es6c01315_si_001.docx]

|  | **East Palestine** | **Marietta** | **UK Staff Day 1** | **UK Staff Day 2** | **NHANES 2015-2016** |
| --- | --- | --- | --- | --- | --- |
| **HEMA** | -- | -- | -- | 8.5 | 1.44 |
| **3HPMA** | 40 | 35.3 | 31.9 | 48.5 | 207 |
| **CEMA** | 33.8 | 86.4 | 45 | 38 | 89.6 |
| **PMA** | -- | -- | -- | -- | 1.14 |
| **BMA** | 2.9 | 4 | 5.1 | 3 | 6.11 |
| **PGA** | 40.9 | 104.6 | 29 | 49.5 | 189 |
| **MHA** | 24.6 | 34.8 | 25.4 | 35.5 | 168.9 |
| **BuAMA** | -- | -- | -- | -- | -- |

**Table S1.** Medians for the analyzed VOC urine metabolites in East Palestine, Marietta, UK staff (Day 1 and 2), and NHANES 2015-2016 data
